# A missense mutation in the *Hspa8* gene encoding heat shock cognate protein 70 causes neuroaxonal dystrophy in rats

**DOI:** 10.3389/fnins.2024.1263724

**Published:** 2024-02-06

**Authors:** Miyuu Tanaka, Ryoko Fujikawa, Takahiro Sekiguchi, Jason Hernandez, Oleta T. Johnson, Daisuke Tanaka, Kenta Kumafuji, Tadao Serikawa, Hieu Hoang Trung, Kosuke Hattori, Tomoji Mashimo, Mitsuru Kuwamura, Jason E. Gestwicki, Takashi Kuramoto

**Affiliations:** ^1^Institute of Laboratory Animals, Graduate School of Medicine, Kyoto University, Sakyo-ku, Kyoto, Japan; ^2^Laboratory of Veterinary Pathology, Graduate School of Veterinary Science, Osaka Metropolitan University, Izumisano, Osaka, Japan; ^3^Department of Pharmaceutical Chemistry and the Institute for Neurodegenerative Diseases, University of California, San Francisco, San Francisco, CA, United States; ^4^Department of Animal Science, Faculty of Agriculture, Tokyo University of Agriculture, Atsugi, Kanagawa, Japan; ^5^Division of Animal Genetics, The Institute of Medical Science, The University of Tokyo, Minato-ku, Tokyo, Japan

**Keywords:** animal model, axon, *Hspa8*, neuroaxonal dystrophy, rat, spheroid

## Abstract

Neuroaxonal dystrophy (NAD) is a neurodegenerative disease characterized by spheroid (swollen axon) formation in the nervous system. In the present study, we focused on a newly established autosomal recessive mutant strain of F344-*kk*/*kk* rats with hind limb gait abnormalities and ataxia from a young age. Histopathologically, a number of axonal spheroids were observed throughout the central nervous system, including the spinal cord (mainly in the dorsal cord), brain stem, and cerebellum in F344-*kk*/*kk* rats. Transmission electron microscopic observation of the spinal cord revealed accumulation of electron-dense bodies, degenerated abnormal mitochondria, as well as membranous or tubular structures in the axonal spheroids. Based on these neuropathological findings, F344-*kk*/*kk* rats were diagnosed with NAD. By a positional cloning approach, we identified a missense mutation (V95E) in the *Hspa8* (heat shock protein family A (Hsp70) member 8) gene located on chromosome 8 of the F344-*kk*/*kk* rat genome. Furthermore, we developed the *Hspa8* knock-in (KI) rats with the V95E mutation using the CRISPR-Cas system. Homozygous *Hspa8*-KI rats exhibited ataxia and axonal spheroids similar to those of F344-*kk*/*kk* rats. The V95E mutant HSC70 protein exhibited the significant but modest decrease in the maximum hydrolysis rate of ATPase when stimulated by co-chaperons DnaJB4 and BAG1 *in vitro*, which suggests the functional deficit in the V95E HSC70. Together, our findings provide the first evidence that the genetic alteration of the *Hspa8* gene caused NAD in mammals.

## 1 Introduction

Neuroaxonal dystrophy (NAD) is a nonspecific, but histologically distinct, inherited neurodegenerative disorder of the central and/or peripheral nervous system. NAD is characterized by swelling of axons (spheroids) (Nardocci and Zorzi, 2013). Ultrastructurally, spheroids appeared to be filled with accumulations of smooth membrane-bound vesicles, membranous lamellae, dense bodies, and other organelles.

Autosomal recessive forms of NAD have been described in humans and animals. In human infantile NAD (INAD; OMIM 256600), ataxia first occurs at ages 1 to 3 and is followed by motor and intellectual disability, cerebellar ataxia, marked truncal hypotonia, pyramidal signs, and early visual disturbances due to optic atrophy (Stankiewicz et al., 2007). Postmortem examination of patients with INAD demonstrated the accumulation of iron in the basal ganglia. Thus, INAD is included in the group of diseases referred to as neurodegeneration with brain iron accumulation (NBIA) (Gregory et al., 2009). INAD is caused by homozygous or compound heterozygous mutations in the *PLA2G6* gene that encodes phospholipase A2, group VI (Khateeb et al., 2006; Morgan et al., 2006): the mitochondrial pathology and degeneration of presynaptic membranes underlie INAD pathology (Beck et al., 2011; Sumi-Akamaru et al., 2015). Mutations in the *PLA2G6* gene have variable phenotypic outcomes, so these different clinical groups have recently been collectively referred to as *PLA2G6*-associated neurodegeneration (PLAN) (Kurian et al., 2008). Mutations in *PLA2G6* have also been reported to be associated with a continuous clinical spectrum ranging from NAD to hereditary spastic paraplegia (HSP), a group of motor neurodegenerative disorders mainly characterized by slowly progressive spasticity and weakness of the lower limbs (Fink, 2013; Ozes et al., 2017; Elsayed et al., 2021; Murala et al., 2021).

In animals, inherited NAD has been identified in various species including dogs (Fyfe et al., 2011; Hahn et al., 2015; Tanaka et al., 2017; Tsuboi et al., 2017; Lucot et al., 2018), cats (Carmichael et al., 1993), horses (Hales et al., 2020), sheep (Letko et al., 2021), and laboratory mice (Saigoh et al., 1999). In dogs, different genes have been identified as causative genes of NAD. For example, a missense mutation in the *PLA2G6* gene has been identified in Papillons (Tsuboi et al., 2017). Missense mutations in the tectonin beta-propeller repeat-containing protein 2 (*TECPR2*) and the vacuolar protein sorting 11 (*VPS11*) genes are associated with NAD in Spanish water dogs and Rottweilers, respectively (Hahn et al., 2015; Lucot et al., 2018). A 3-bp deletion in the mitofusin 2 (*MFN2*) was also found in a breeding colony of the laboratory dogs (Fyfe et al., 2011).

In mice, *Pla2g6* knockout (KO) mice and *Pla2g6*-mutated mice have been established. In addition, an intragenic deletion in the ubiquitin carboxy-terminal hydrolase isozyme (*Uchl1*) causes NAD in the gracile axonal dystrophy (*gad*) mice which show the axonal degeneration with progressive sensory-motor ataxia (Saigoh et al., 1999; Shinzawa et al., 2008; Wada et al., 2009; Onishi et al., 2013; Sumi-Akamaru et al., 2015). Genes identified as causative in these cases of animal NAD are involved in autophagy, membrane trafficking, mitochondrial metabolism, and proteolysis, which suggests that defects in these functions in neurons play critical roles in the development of NAD. Thus, identification of genes involved in the hereditary NAD in animal models can contribute to an understanding of the pathomechanisms underlying NAD and hereby lead to the development of diagnosis and treatment of NAD in humans as well as domestic animals.

The HSPA8/HSC70 protein (Heat shock cognate 71 kDa protein), encoded by *Hspa8* (heat shock protein family A (Hsp70) member 8) gene, is a constitutively expressed molecular chaperone that is critical for protein quality control in cells (Stricher et al., 2013; Zuiderweg et al., 2017). HSC70 plays a pivotal role in folding and refolding, facilitates protein trafficking across membranes, and targets proteins for degradation (Stricher et al., 2013). HSC70 is involved in many physiological functions, such as autophagy (Bonam et al., 2019), clathrin-mediated endocytosis (McMahon and Boucrot, 2011), and regulation of viral infections (Wang et al., 2020). HSC70 has also been known to be associated with many pathological conditions, including cancers (Tian et al., 2018; Martyna et al., 2019; Chen et al., 2020; Liu et al., 2021), viral infections (Dupzyk and Tsai, 2018; Wang et al., 2020; Zhu et al., 2020; Chailangkarn et al., 2021), and neurological disorders (Sirtori et al., 2020). Although alterations in expression levels of HSC70 are associated with various diseases, no mutation of the *Hspa8* gene is known in hereditary disorders either in humans or animals, possibly because of its pivotal role in the protein quality control in cells.

Rats showing hind limb ataxia appeared in F2 progeny of F344-*Sv2a^m1Kyo^* rats. The F344-*Sv2a^m1Kyo^* rats were generated by a gene-driven N-ethyl-N-nitrosourea mutagenesis (Mashimo et al., 2008) and carried a missense mutation (L174Q) in the synaptic vesicle glycoprotein 2A (*Sv2a*) gene on a F344/NSlc background (Tokudome et al., 2016). Even after removing the *Sv2a^m1Kyo^* missense mutation, rats showing hind limb ataxia appeared in subsequent generations. Hind limb ataxia was found at approximately 6–7 weeks of age and rapidly worsened. The affected rats wasted away in a few weeks after onset. The hind limb ataxia phenotype is inherited in an autosomal recessive manner. We named a causative gene of this phenotype *kk* after the initials of the first person who discovered the affected rats.

In the present study, we established an F344-*kk*/*kk* rat strain and characterized the histopathology of this strain. To identify the *kk* mutation, we used a positional cloning approach and found a missense mutation in the *Hspa8* gene of the F344-*kk*/*kk* rat genome. Next, we developed the knock-in rats using the CRISPR-Cas system to prove that the mutation was causative of the NAD in rats. Finally, we characterized the mutant protein *in vitro*.

## 2 Materials and methods

### 2.1 Ethical use of animals

All animal experiments were approved by the Animal Research Committees of Kyoto University, Osaka Metropolitan University, and Tokyo University of Agriculture and were conducted according to their regulations on animal experimentation.

### 2.2 Animals

F344-*kk/kk* rats were obtained from the National BioResource Project for the Rat (NBRP-Rat, Kyoto, Japan) (Serikawa et al., 2009). BN/SsNSlc and F344/NSlc rats were purchased from Japan SLC, Inc., (Hamamatsu, Shizuoka, Japan).

### 2.3 Characterization of the gaits

Lengths and widths of gaits in *kk*-homozygous (*n* = 4) and wild-type (WT) (*n* = 4) female rats were examined at 9 weeks of age (Teunissen et al., 2001; Nishitani et al., 2020). Foot pads of the hind paws were immersed in the black ink and the rats were placed on the white absorbing paper (12 cm × 100 cm). Step lengths were measured for the right and left legs. Step widths were measured for every successive step.

### 2.4 Histopathology and transmission electron microscopy

Rats (3, 5, 7, 8, 9, and 10 weeks of age) were euthanized under isoflurane anesthesia. We examined a total 112 rats for histopathology (homozygous; *n* = 79 and WT; *n* = 33). Tissue samples from the central nervous system (CNS) were fixed in 10% neutral buffered formalin, and embedded in paraffin. Sections of 4 μm thick were cut and stained with hematoxylin and eosin (HE). We also counted the number of spheroids (exceedingly 5 μm in diameter) in the transverse sections of the cervical (C5 level) and lumber (L1–2 level) spinal cord at 10 weeks of age by microscopic observation. The areas of 2.37 mm^2^ (high-power fileld) in the dorsal cord of the spinal cord from five different animals were evaluated in each experimental group. The data are presented as the number of spheroids/mm^2^. For transmission electron microscopy (TEM), two formalin-fixed tissues of *kk*-homozygous rats at 5 and 10 weeks of age were stored in 2.5% glutaraldehyde in 0.1 M phosphate buffer (pH 7.4), post-fixed with 1% osmium tetraoxide at 4°C overnight and embedded in epoxy resin. Ultrathin sections were stained with uranyl acetate and lead citrate and examined with a Hitachi H-7500 electron microscope (Hitachi, Tokyo, Japan).

### 2.5 Immunohistochemistry

We conducted immunohistochemistry (IHC) using formalin-fixed paraffin sections of the lumbar spinal cord from 10 weeks of age. For IHC, we used the primary antibodies listed in Supplementary Table S1. After dewaxing and pretreatment, immunohistochemical staining was performed using a HISTOSTAINER 36A (Nichirei Biosciences, Tokyo, Japan). Sections were treated with 5% skimmed milk in phosphate-buffered saline for 15 min and reacted with each primary antibody for 1 h at room temperature. After incubation in 3% H_2_O_2_ for 15 min, application of horseradish peroxidase-conjugated secondary antibodies (Histofine Simple Stain MAX PO; Nichirei Biosciences) was performed for 1 h. Signals were visualized with 3,3′-diaminobenzidine (DAB Substrate Kit; Nichirei Biosciences).

### 2.6 Genetic mapping

Because *kk*/*kk* homozygous rats died before sexual maturity, we used *kk*/+ heterozygous rats for breeding. We intercrossed *kk*/+ heterozygous (BN/SsNSlc × F344-*kk*/+)F1 rats to produce F2 intercross or backcrossed to F344-*kk*/+ rats to produce backcross progeny. Genotyping for the *kk* locus was performed by observation of rats exhibiting abnormal gaits and wasting postures by 10 weeks of age. Only *kk*/*kk* homozygous rats were used for genetic mapping of the *kk* gene. To localize the *kk* locus to a specific chromosomal region, we performed genome-wide scanning on DNA samples from 22 *kk*/*kk* homozygous rats using a panel of 106 simple sequence length polymorphism (SSLP) markers that covered all autosomal chromosomes (Chrs) (Supplementary Figure S1). To narrow down the *kk* locus, we additionally used a SSLP marker (*D8Rat46*) and a SNP marker (J683662). Genotyping of the SNP marker was performed by direct sequencing of PCR products amplified with the following primer set; 5′-AGGCTCCTGAGCAAGTTCAA-3′ and 5′-TGCAGTCCTAGGTATCCCTTT-3′.

### 2.7 Reverse transcription–polymerase chain reaction and sequencing

Total RNA was isolated from brains using ISOGEN II (Nippon Gene, Tokyo, Japan). reverse transcription–polymerase chain reaction (PCR) and direct sequencing of PCR products were carried out as described previously (Kuramoto et al., 2011). Details of primers are listed in Supplementary Table S2. The PCR products overlapped each other and spanned the entire coding sequence of the rat *Hspa8* gene. All sequencing was performed by Macrogen Japan Corporation (Kyoto, Japan).

### 2.8 Genotyping of the T to A substation of the *Hspa8* gene

Genotyping of the T to A substation of the *Hspa8* gene was performed by the Amp-FTA method (Nakanishi et al., 2009). Templates were prepared on the FTA card and amplified with the following primer set: 5′-ATTAAATATGGGACATTGCTTC-3′ and 5′-CCTTTGTATTCGACTTGGAC-3′. The substitution was detected by Cycleave PCR™ Assay (Takara Bio Inc., Kusatsu, Shiga, Japan), in which fluorescence-labeled DNA–RNA chimeric probes were used. Sequences of the probes were as follows: 5′-ATGGTGG(rA)GA-3′ for the mutant allele and 5′-TGGTGGT(rG)AA-3′ for the WT allele.

### 2.9 Development of genetically modified rats by genome editing

Genome editing by CRISPR-Cas was performed as described previously (Yoshimi et al., 2016). Guide RNAs (gRNAs) were designed by Optimized CRISPR Design (crispr.mit.edu) and synthesized by Integrated DNA Technologies, Inc., (Coralville, IA, United States). Long ssODNs (lsODNs) were prepared using a LsODN Preparation Kit (Biodynamics Laboratory Inc., Tokyo, Japan). Cas9 protein was purchased from Integrated DNA Technologies. Pronuclear-stage embryos of F344/Jcl (CLEA Japan, Inc., Tokyo, Japan) rats were produced by natural mating. The oviducts of female rats with vaginal plugs were removed after euthanasia by CO_2_ and cervical dislocation, and embryos were flushed out from the ampullae with culture medium. Cas9 protein, gRNAs, and lsODN were introduced into the embryos using a super electroporator NEPA 21 (NEPA GENE Co., Ltd., Ichikawa, Chiba, Japan). Embryos that developed to the two-cell stage were transferred into the oviducts of pseudopregnant females that were anesthetized using isoflurane. Offspring were genotyped by the Amp-FTA method with the following primer set: 5′-CGGGTTTCAGAGATGGAAGA-3′ and 5′-ATTTTCATTGACAGGTCCGG-3′ and Ampdirect Plus buffer (Shimadzu Corporation, Kyoto, Japan). Founder rats were mated with F344/Jcl rats and F1 heterozygous rats were intercrossed to obtain F2 progeny.

### 2.10 Western blot

Thoracic spinal cords of *Hspa8* knock-in (KI) homozygous and WT rats at 9 weeks of age were removed and homogenized in a cell lysis reagent (CelLytic MT, Sigma Aldrich, St. Louis, MO, United States) with proteinase inhibitor cocktail (Nacalai tesque, Kyoto, Japan). The supernatants were collected after centrifugation at 13,000 ×*g* for 10 min and protein concentrations were determined by an absorption spectrometer using the Bradford protein Assay (Bio-Rad Laboratories, Hercules, CA, United States). The supernatants were boiled for 5 min with SDS sample buffer (Cosmo Bio, Tokyo, Japan) with 5% 2-mercaptoethanol (Bio-Rad Laboratories). Samples were separated on 5–20% gradient polyacrylamide gels (ATTO Corporation, Tokyo, Japan) and transferred to polyvinylidene difuoride (PVDF) membranes (Bio-Rad Laboratories). Membranes were incubated overnight at 4°C with the following antibodies: rabbit monoclonal anti-HSC70 (clone EP1531Y, ab51052, 1:2,000; Abcam, United Kingdom) and mouse monoclonal anti-β-actin (clone AC-15, A54411, 1:30,000; Sigma Aldrich). After washing, the membranes were treated with peroxidase-conjugated secondary antibody (Histofine Simple Stain MAX PO; Nichirei Biosciences) for 30 min (for β-actin) or 1 h (for HSC70) at room temperature. Signals were visualized with ECL-prime (GE Healthcare, United Kingdom) and quantified with a luminescent image analyzer (LAS-4000; GE Healthcare). β-actin was used as an internal control.

### 2.11 Protein expression and purification

Human WT and V95E HSC70 (HSPA8) were expressed and purified as previously described (Rauch et al., 2016; Johnson et al., 2022). In brief, inoculated bacterial cultures were grown to OD600 = 0.6 and cooled to 20°C. At this point, protein expression was induced with 200 μL IPTG and cultures were incubated with shaking overnight. Cells were harvested and the resulting pellet was either frozen at-80°C or carried forward immediately to purification. Cell pellets were lysed by sonication and cleared lysate was applied to Ni-NTA resin (Thermo Fisher Scientific, Waltham, MA, United States). The Ni-NTA elution was treated with TEV protease (MacroLab; University of California, Berkeley) to cleave the 6His-tag and the cleavage product was further purified by an ATP-agarose (Sigma Aldrich) column. DnaJB4 was expressed and purified as previously reported (Rauch et al., 2016). Inoculated bacterial cultures were grown, induced, and harvested as above. Cell pellets were lysed by sonication and cleared lysate was applied to Ni-NTA resin (Thermo Fisher Scientific). The Ni-NTA elution was treated with TEV protease (MacroLab; University of California, Berkeley) to cleave the 6His-tag and the cleavage product was further purified by a Superdex S200 (GE Healthcare) size exclusion column. BAG1 was expressed and purified as described elsewhere (Rauch et al., 2016). Briefly, cultures were handled as above and then cells were lysed by sonication and lysate was applied to a HisPur Ni-NTA resin (Thermo Fisher Scientific). The Ni-NTA elution was treated with TEV protease (MacroLab; University of California, Berkeley) and the cleavage product was subsequently purified by ion-exchange chromatography (MonoQ, GE Healthcare).

### 2.12 ATPase activity assay

ATP hydrolysis activity was assessed in a 96-well plate format (Fisher #12565501) using a malachite green assay that measures the generation of inorganic phosphate upon ATP hydrolysis as previously described (Chang et al., 2008; Rauch and Gestwicki, 2014; Rauch et al., 2016; Taylor et al., 2018). In short, 1 μM HSC70 was titrated with increasing concentrations of the co-chaperone of interest (DnaJB4 or BAG1). Reactions were initiated by the addition of excess ATP and incubated at 37°C for 1 h. Malachite green reagent was then added to each well and reactions were immediately quenched with sodium citrate. Absorbance was measured at 620 nm using a SpectraMax M5 plate reader (Molecular Devices). Experiments were performed in triplicate. DnaJB4 data were fit by the Michaelis–Menten equation to derive pseudo-K_m_ and pseudo-V_max_ values. DnaJB4 and BAG1 titration data were normalized as percentages by taking the lowest and highest value in each subcolumn as 0 and 100%, respectively, in GraphPad Prism 9 (GraphPad Software, Boston, MA, United States) software.

### 2.13 Luciferase refolding assay

The luciferase refolding assay was performed in a 96-well format (Corning #3912) as reported previously (Rauch and Gestwicki, 2014; Rauch et al., 2016; Taylor et al., 2018). Briefly, Renilla luciferase (0.5 mg/mL, Promega, Madison, WI, United States) was denatured in buffer A (25 mM HEPES; pH 7.2, 50 mM potassium acetate, and 5 mM dithiothreitol) containing 8 M guanidine hydrochloride (GuHCl) at room temperature for 120 min. The denatured protein was diluted 1:40 in buffer A and placed on ice for 20 min. Refolding reactions were then prepared by adding 2 μL of the denatured luciferase stock into 48 μL of refolding buffer (28 mM HEPES; pH 7.6, 120 mM potassium acetate, 12 mM magnesium acetate, 2.2 mM dithiothreitol, 0.1 mM ATP, 8.8 mM creatine phosphate, and 35 U/mL creatine kinase) containing HSC70 (1 μM) and varying concentrations of the indicated co-chaperone (DnaJB4 and/or BAG1). Refolding reactions were initiated by the addition of excess ATP (1 mM) and incubated at 37°C for 1 h. After this incubation, SteadyGlo (Promega) reagent was added and the luminescence signal was immediately measured using a SpectraMax M5 plate reader. Titration data were normalized as percentages by taking the lowest and highest luminescence values as 0 and 100%, respectively, in GraphPad Prism 9 software. DnaJB4 data were fit by the Michaelis–Menten equation to derive half-maximal effect values and no statistically significant difference (*p* > 0.05) was observed in the WT HSC70 vs. HSC70 V95E results. Experiments were repeated on three separate protein samples (biological replicates) and each experiment was separated into three wells (technical triplicates; total *n* = 9).

### 2.14 Fluorescence polarization assay

FP saturation assays were carried out as previously described (Rauch and Gestwicki, 2014; Rauch et al., 2016). Briefly, 20 nM N6-(6-Amino)hexyl-ATP-5-FAM (ATP-FAM) (Jena Bioscience) was incubated with a titration of HSC70 in black, round-bottom, low-volume, 384-well plates (Corning #4511) for 30 min at room temperature. Fluorescence polarization was measured (excitation, 485 nm; emission, 535 nm) using a SpectraMax M5 plate reader. Experiments were performed in triplicate and data were fit using a sigmoidal dose–response (variable slope) curve in GraphPad Prism 9. Data was transformed as (x = log [HSC70 (μM)]).

### 2.15 Statistical analysis

Data are expressed as the mean ± SD. Data were analyzed using GraphPad Prism 9. The statistical significance of differences among multiple groups was determined by two-way ANOVA with Bonferroni’s post-hoc test. Comparisons between the two groups only were determined by Student’s or Welch’s *t*-test. *p* < 0.05 were considered statistically significant (see figure legends).

## 3 Results

### 3.1 Clinical symptoms of F344-*kk*/*kk* rats

**Figure 1:**
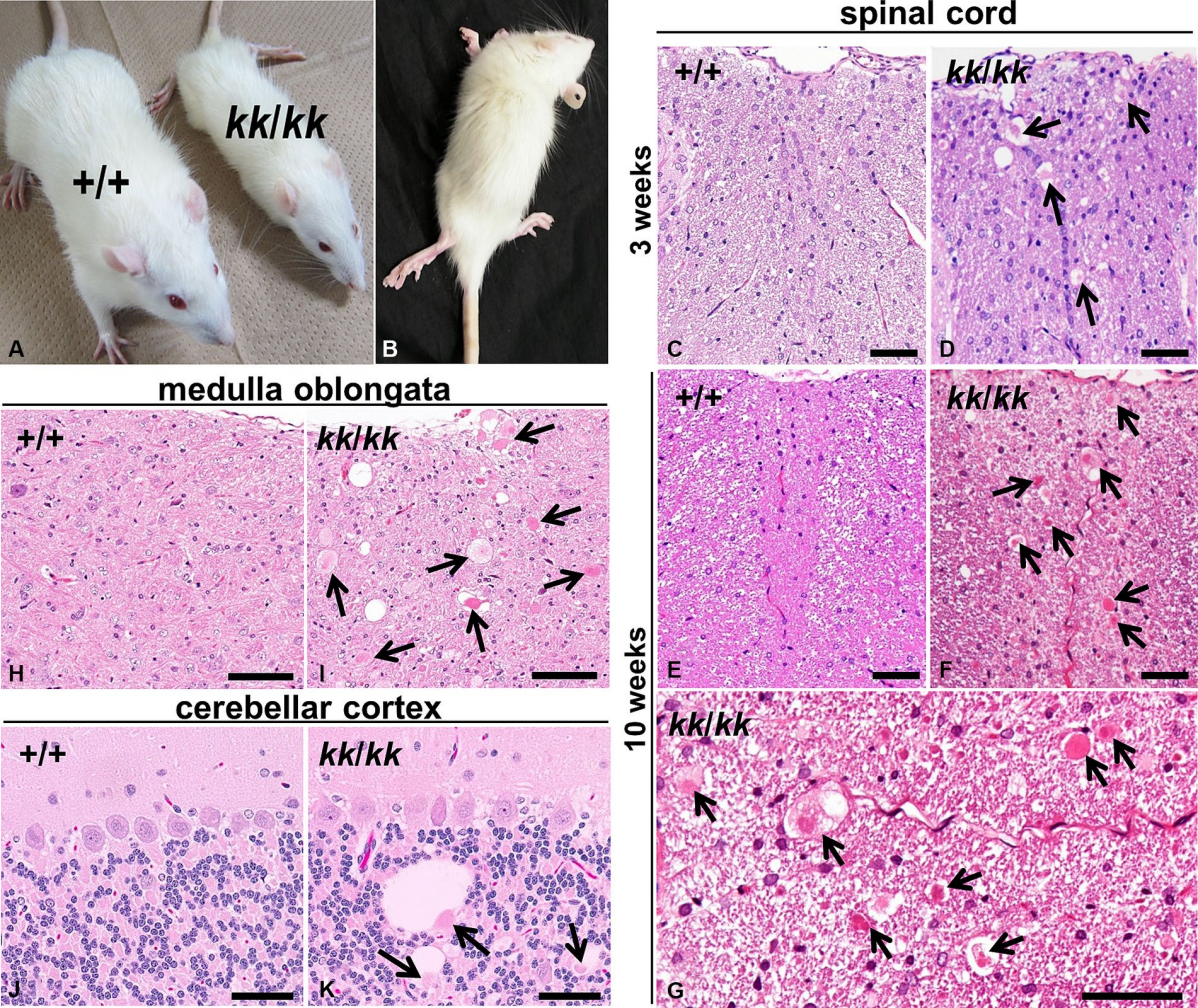
Neuroaxonal dystrophy in F344-*kk/kk* rats. **(A)** Gross appearances of a rat homozygous *kk* (*kk*/*kk*) and its littermate wild-type (WT) (+/+) rats at 9 weeks of age. The *kk*/*kk* homozygous rat exhibited small body size and failed to walk normally. **(B)** Hind limbs of a *kk*/*kk* homozygous rat (10 weeks of age). Marked hind limb ataxia and wasting were observed. **(C–K)** Histopathology of the CNS in F344-*kk/kk* rats at 3 weeks **(C,D)**, 9 weeks **(H–K)**, and10 weeks **(E–G)** of age. Histopathology of the dorsal cord of the lumbar spinal cord in the WT **(C,E)** and *kk*/*kk* homozygous **(D,F,G)** rats. Histopathology of the medulla oblongata in the WT **(H)** and *kk*/*kk* homozygous **(I)** rats. Histopathology of the cerebellar cortex in the WT **(J)** and *kk*/*kk* homozygous **(K)** rats. Arrows indicate axonal spheroids. H and E. Bars: 50 μm **(C–G,J,K)** and 100 μm **(H,I)**.

To find the onset of clinical symptoms of the F344-*kk*/*kk* rats, we examined when abnormal gaits appeared. At 3 weeks of age, the *kk*/*kk* homozygous rats showed an unsteady hindlimb gait compared with control rats (Supplementary Video S1). Almost all *kk*/*kk* rats (94% of *kk*/*kk* rats) developed abnormal gaits appearing in the hind limbs between 7 and 8 weeks of age. The abnormal gaits were characterized by the significantly shorter step lengths: the step lengths of the homozygous rats were shorter than the WT rats (7.7 ± 0.37 cm vs. 10.0 ± 1.43 cm, *p <* 0.001) but the step widths were not different between the homozygous and WT rats (4.3 ± 0.23 cm vs. 4.0 ± 0.51 cm, *p* = 0.13). The *kk*/*kk* homozygous rats exhibited the complete ataxia of the limbs and stooping position, hindlimb splay/extension, and eventually fell into a complete prone position (Figures 1A,B). After the onset of the abnormal gaits, *kk*/*kk* rats could not survive longer than 4 weeks. We observed mortalities of male (*n* = 8) and female (*n* = 5) *kk*/*kk* rats for 12 weeks and found that the mortalities were not different between male and female rats (9.5 ± 0.69 vs. 10.2 ± 1.10 weeks of age, *p* = 0.135). The average mortality of the *kk*/*kk* rats was 9.79 ± 0.89 weeks of age. No WT rat died during the 12-weeks observation periods. Additionally, *kk*/*kk* rats exhibited significantly smaller body size than the WT rats from 3 weeks of age (Figure 1A; Supplementary Figure S2).

### 3.2 Rats with the homozygous *kk* mutation exhibited neuroaxonal dystrophy

To examine histopathological alterations in the CNS, we performed light microscopic observation. We found dystrophic swollen axons throughout the CNS from at least 3 weeks of age in the *kk*/*kk* homozygous rats (Figure 1). The axonal spheroids varied in size (approximately 5–50 μm) and were heterogeneous in morphology: they showed a homogeneous or granular appearance with or without clefts and vacuoles (Figure 1G). Such axonal swellings were not observed at all in the WT rats. In the spinal cord, axonal spheroids were observed in both white and gray matter, located mainly in the dorsal cord (fasciculus gracilis and dorsal corticospinal tract) (Figures 1D,F). In gray matter, lesions locate in the dorsal horn rather than the ventral horn. The spinal cord lesions were more severe in the posterior than the anterior of the spinal cord; the latter part of thoracic, lumber, and sacral parts exhibited more severe lesions than the cervical parts of the spinal cord (Supplementary Figure S3). In the brain stem, numerous large axonal spheroids were observed from at least 3 weeks of age, especially in the nucleus gracilis, nucleus cuneatus, and nucleus cuneatus accessorius of the medulla oblongata (Figure 1I). In the cerebellum, cerebellar white matter, cortex (predominantly in the granular cell layer), and the inferior and middle cerebellar peduncle were primarily affected. Some spheroids were associated with large vacuoles in the granular layers (Figure 1K). Based on these neuropathological findings, the F344-*kk*/*kk* rats were histopathologically diagnosed with NAD.

### 3.3 Ultrastructural and immunohistochemical findings of NAD in F344-*kk/kk* rats

**Figure 2:**
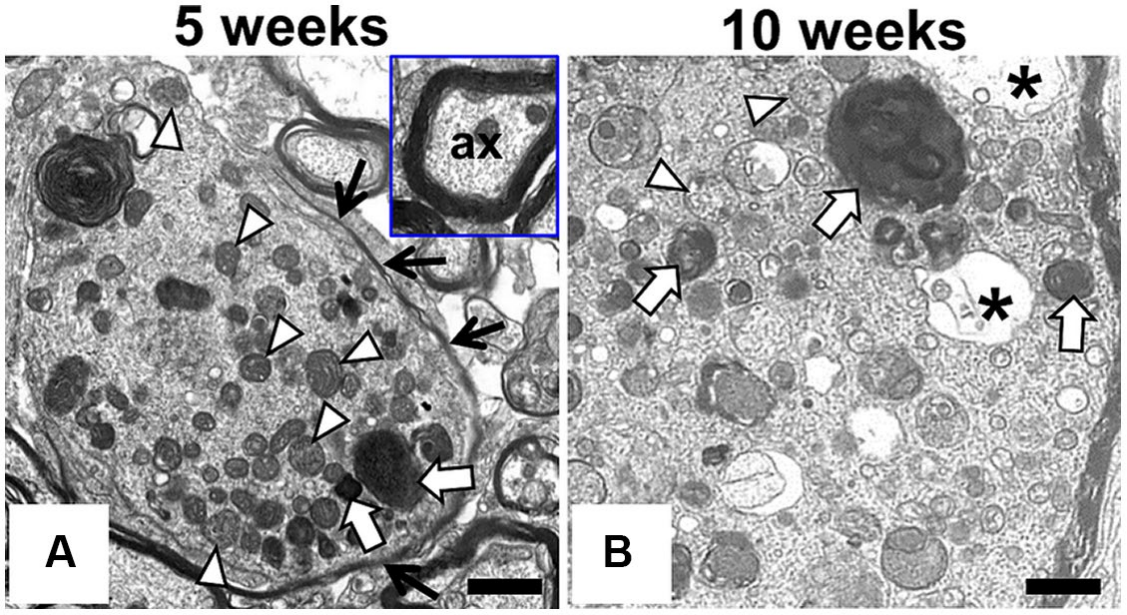
Ultrastructural findings in the swollen axon of F344-*kk/kk* rat. Transmission electron microscopy (TEM) from the dorsal cord of the lumber spinal cord in the *kk*/*kk* homozygous rat at 5 weeks **(A)** and 10 weeks of age **(B)**. Axonal spheroids are filled with dense bodies (white arrows), densely packed abnormal mitochondria (white arrowheads), membranous or tubular, filamentous materials, and edematous vacuoles (black asterisks). Black arrows show a part of myelin sheath. Inset: non-swollen axon (ax) with normal myelin sheath. Bars: 1 μm.

To examine morphology of the swollen axons of F344-*kk/kk* rats in detail, we performed TEM experiments on the dorsal cord of the lumber spinal cord in the *kk*/*kk* homozygous rats (Figure 2). We found the accumulation of electron-dense bodies (Figure 2: white arrows), degenerated or swollen abnormal mitochondria (Figure 2: white arrowheads), membranous or tubular structures, edematous vacuoles (Figure 2: asterisks), and neurofilaments (NFs) in the swollen axons (Figure 2). Swollen axons sometimes lacked a myelin sheath. These findings were consistent with the ultrastructural observations previously reported in patients and animals with NAD (Yagishita and Kimura, 1974; de Leon and Mitchell, 1985; Beck et al., 2011; Tanaka et al., 2017).

**Figure 3:**
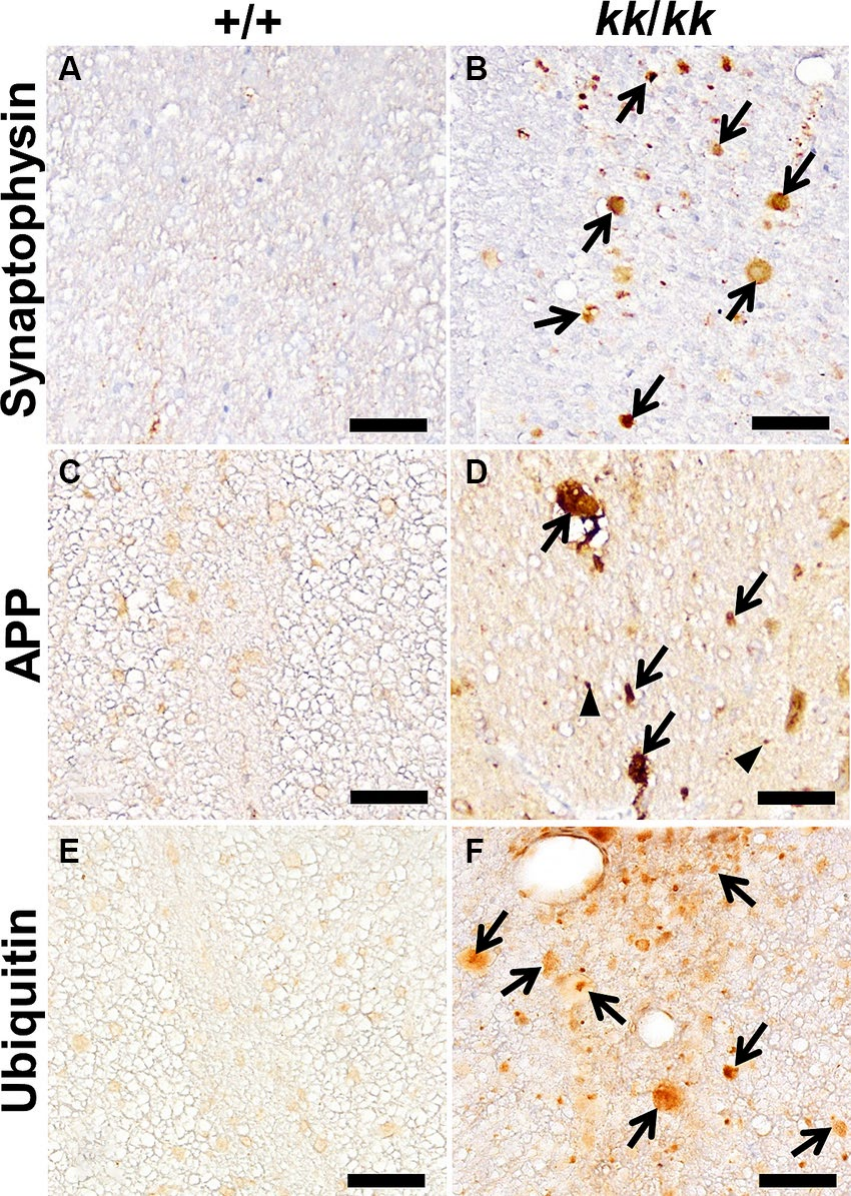
Immunohistochemical findings in the axons of F344-*kk/kk* rat. Immunohistochemistry (IHC) for Synaptophysin **(A,B)**, APP **(C,D)**, and ubiquitin **(E,F)** of the dorsal cord of the lumbar spinal cord in the wild-type (WT) (+/+) **(A,C,E)** and *kk/kk* homozygous **(B,D,F)** rats at 10 weeks of age. Arrows indicate axonal spheroids that show strong or abnormal immunoreactivity for each protein. Arrowheads indicate APP immunoreactivity in non-swollen axons. No accumulation of these proteins is observed in the axons of the WT rats. Bars: 50 μm.

To characterize the spheroids and predict the pathogenesis of NAD in F344-*kk*/*kk* rats, we performed IHC analysis (Figure 3). Axonal spheroids were strongly positive for synaptophysin, a synapse-associated glycoprotein on presynaptic vesicles (Figure 3B). The amyloid β precursor protein (APP) is thought to be an effective marker for axonal injury (Gentleman et al., 1993; Hayashi et al., 2015). Although the APP was not accumulated in axons of the WT rats (Figure 3C), it was accumulated in swollen axons and even in some non-swollen axons in F344-*kk/kk* rats (Figure 3D). This finding indicated that both swollen and non-swollen axons were injured in the F344-*kk/kk* rats. In addition, the spheroids were also positive for neurofilament (neuron-specific intermediate filaments) proteins (Supplementary Figure S4) and ubiquitin (Figure 3F), an important factor for post-translational modifications (Lee et al., 1987; Yuan and Nixon, 2021). These proteins were not accumulated within axons of the WT rats.

### 3.4 Neurological syndrome was associated with a missense mutation in the *Hspa8*, a gene encoding heat shock cognate protein 70

**Figure 4:**
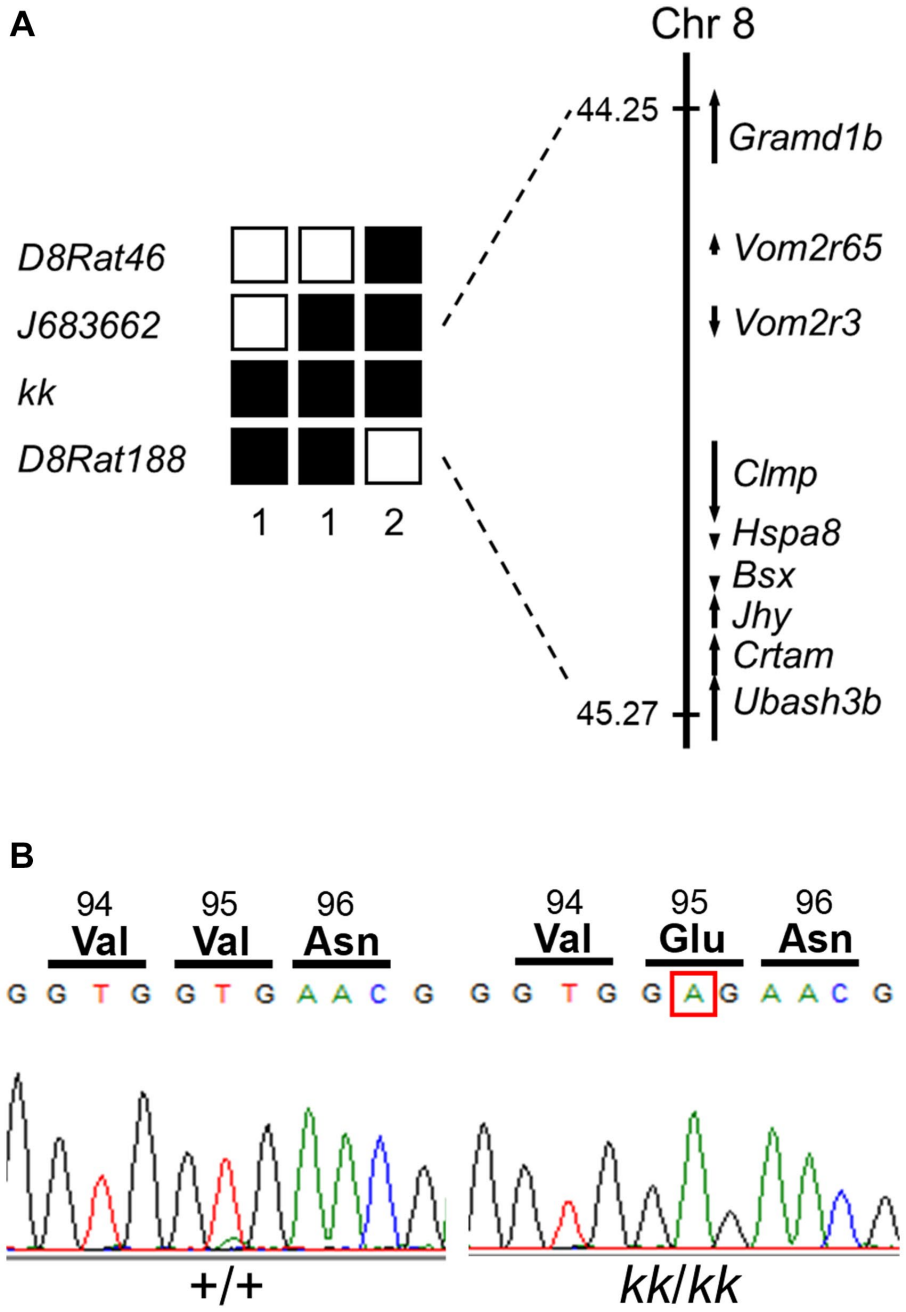
Identification of a missense mutation in *Hspa8* gene of the F344-*kk*/*kk* rat. **(A)** Left; haplotypes of *kk*/*kk* homozygous F2 and backcross progeny carrying the recombinant chromosome between *D8Rat46* and *D8Rat188*. The filled boxes represent rats homozygous for the F344 allele, while the open boxes represent rats heterozygous or homozygous for the BN allele. The number of progeny for each haplotype is described below the haplotypes. **(A)** Right; physical map of the *kk* locus. The *kk* locus was mapped in the 1.02-Mb genomic region between *J683662* and *D8Rat188*. Nine genes were mapped within the *kk* locus. Physical positions of SSLP markers and genes were referred to in Rnor_6.0. **(B)** A nucleotide substitution from T to A at nucleotide 284 of the coding sequence of *Hspa8* in *kk*/*kk* homozygous rats. The substitution converted valine to glutamate at the 95th amino acid position.

To identify the causative mutation of the *kk*, we performed a positional cloning approach. We obtained 171 *kk*/*kk* homozygous rats from F2 intercross and 87 *kk*/*kk* homozygous rats from backcross progeny. Genome-wide scanning using 22 *kk*/*kk* rats mapped *kk* to the rat Chr 8. Fine mapping using the remaining *kk*/*kk* progeny mapped the *kk* between *J683662* and *D8Rat188* (Figure 4A). The *kk* was physically mapped within a ~ 1.02-Mb region defined by *J683662* and *D8Rat188*, in which nine genes were included. We searched the Rat Genome Database for genes that were expressed in the CNS and found that *Clamp* (CXADR-like membrane protein), *Hspa8* [heat shock protein family A (Hsp70) member 8], *Bsx* (brain-specific homeobox), *Jhy* (junctional cadherin complex regulator), and *Ubash3b* (ubiquitin associated and SH3 domain containing, B) were expressed in the CNS.

To find the mutation, we sequenced coding regions of these candidate genes of F344-*kk*/*kk* rats. Although no mutation was found in the *Clamp*, *Bsx*, *Jhy*, or *Ubash3b* genes, we found a nucleotide substitution from T to A at nucleotide 284 of the coding sequence (c.284 T > A) of *Hspa8* (Figure 4B). The substitution was predicted to change valine (Val) to glutamate (Glu) at amino acid 95 located in the nucleotide-binding domain (NBD) of the rat HSC70 protein (Figure 4B; Supplementary Figure S5). Missense prediction analysis with the PROVEAN software predicted that Val95Glu (V95E) was deleterious (score: −3.629) (Choi et al., 2012). All of the 171 *kk*/*kk* progeny were homozygous for the V95E missense mutation.

### 3.5 *Hspa8* knock-in rats exhibited hind limb ataxia and NAD

**Figure 5:**
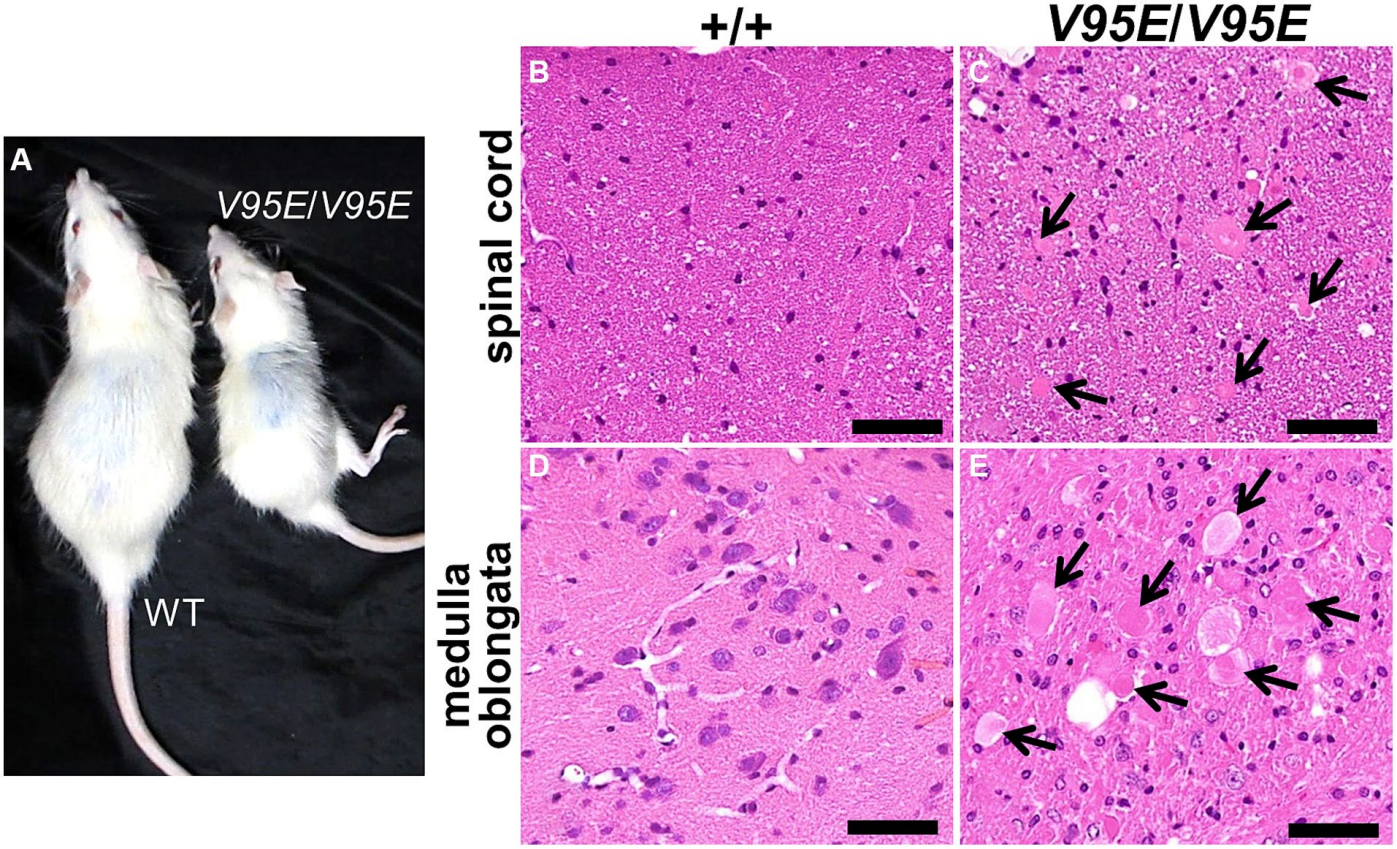
Phenotypes of *Hspa8*-KI homozygous rats. **(A)** Gross appearances of *Hspa8*-KI homozygous (*V95E*/*V95E*) and the wild-type (WT) rats at 11 weeks of age. *Hspa8* KI rats exhibit small body size and hind limb ataxia. **(B–E)** Histopathology of the dorsal cord of the spinal cord **(B,C)** and medulla oblongata **(D,E)** in *Hspa8* KI homozygous (*V95E*/*V95E*) **(C,E)** and WT (+/+) **(B,D)** rats. Arrows indicate axonal spheroids. H and E. Bars: 50 μm.

**Figure 6:**
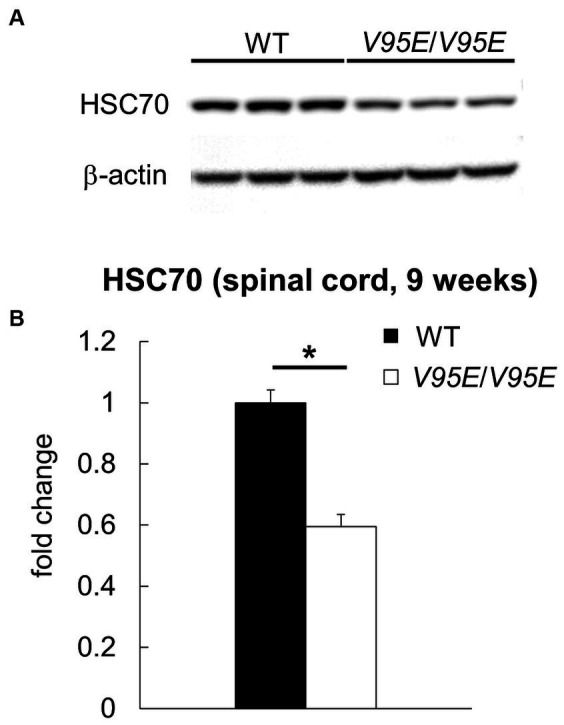
The protein expression of HSC70 in *Hspa8*-KI homozygous rats. **(A,B)** Western blot analysis for HSC70 in the thoracic spinal cord of 9-week-old wild-type (WT) and *Hspa8*-KI homozygous (*V95E*/*V95E*) rats (*n* = 3 in each group). The expression level of HSC70 (71 kDa) is significantly decreased in the spinal cord of *Hspa8*-KI homozygous rats. β-actin was used for the internal control. Data are expressed as fold change from control (WT) **(B)**. ^*^p < 0.05 versus WT by Welch’s *t*-test.

To examine whether the V95E missense mutation caused NAD in F344-*kk/kk* rats, we produced *Hspa8* knock-in (KI) rats that harbored the mutation. Knocking in of the mutant allele was performed by lsODN-mediated knock-in with the CRISPR-Cas9 system (Yoshimi et al., 2016; Supplementary Figure S6). We obtained 24 pups from pseudopregnant rats that electroporated embryos were transferred into. Direct sequencing analyses revealed that 4 rats were potential founders. We developed two *Hspa8*-KI rat strains. *Hspa8*-KI rats, homozygous for the V95E mutation, exhibited abnormal gaits of the hind limbs after 5 weeks of age and deteriorated with increasing age (Figure 5A). Heterozygous and WT rats did not show these clinical signs. The expression level of HSC70 protein was significantly decreased in the spinal cord of *Hspa8*-KI homozygous rats compared with the WT rats, which may contribute to the disease phenotype in the *Hspa8*-mutant rats (Figure 6). Histopathologically, the *Hspa8*-KI homozygous rats exhibited spheroids in the CNS, which was very similar to those of F344-*kk*/*kk* rats (Figures 5B–E). Thus, we concluded that the V95E missense mutation of the *Hspa8* gene was the causative factor of NAD in rats.

### 3.6 Characterization of the HSC70 V95E protein

**Figure 7:**
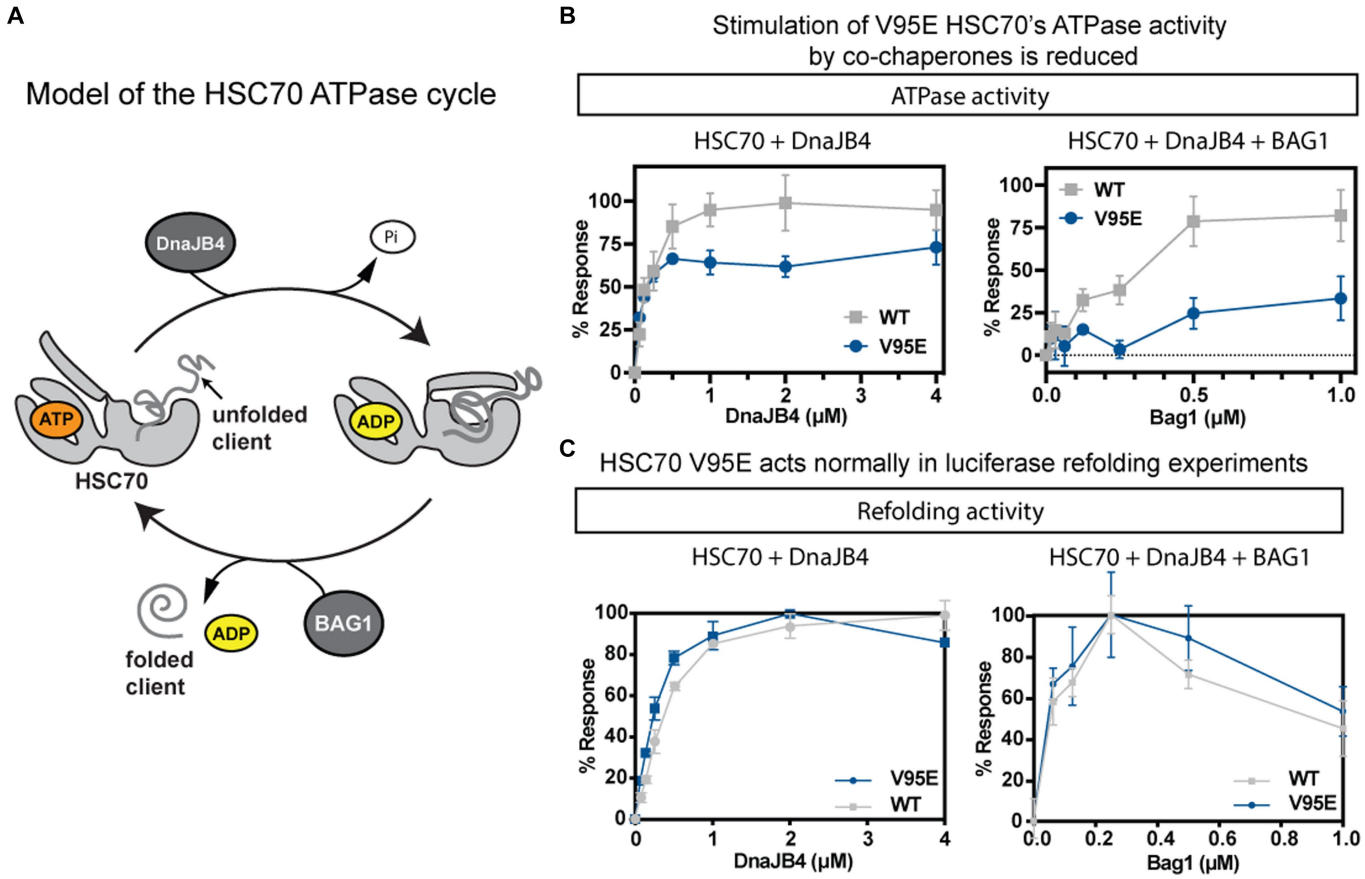
The biochemical activity of purified HSC70 is modestly affected by V95E mutation. **(A)** Schematic of HSC70’s ATPase cycle. HSC70 undergoes rounds of nucleotide hydrolysis, in collaboration with J-domain proteins, such as DnaJB4, and nucleotide exchange factors (NEFs), such as BAG1. **(B,C)** Comparison between the activities of the wild-type (WT) and V95E mutant in ATPase **(B)** and luciferase refolding **(C)** assays. In both assays, activity was measured in the presence of the co-chaperones DnaJB4 (left) or the combination of DnaJB4 and BAG1 (right). Results are the average of three independent experiments performed in technical triplicates (*n* = 9) and error bars represent standard deviation (SD).

HSC70 is composed of two domains: the N-terminal NBD and the C-terminal substrate-binding domain (SBD). The NBD, where the V95E mutation is located, hydrolyzes ATP and this activity is coupled to the binding and release of its peptide/protein substrates in the SBD. The SBD itself may be further distilled to an α-helical lid and a β barrel that directly interacts with its substrates (Figure 7A). Hydrolysis of ATP in the NBD is known to cause a conformation change that “closes” the lid and strengthens affinity for misfolded proteins (termed “clients”); therefore, the V95E mutation may affect ATP hydrolysis or functional coupling between the NBD and SBD. Furthermore, this hydrolysis cycle is assisted by co-chaperone proteins, including J-domain proteins (JDPs), which catalyze ATPase activity, and nucleotide exchange factors (NEFs), including those which reset the nucleotide cycle to promote iterative ATP binding, hydrolysis, and ADP release from the NBD (Figure 7A). Thus, another outcome of the V95E mutation may be disruption in the functional interaction of HSC70 with its co-chaperone partners.

To generate a working hypothesis for how the V95E mutation might impact HSC70 or its interactions with co-chaperones, we mapped the location of the mutation onto a structure of the NBD of human HSC70 (pdb 4H5T). First, we noticed that the V95 position is far from residues known to be critical for nucleotide binding or hydrolysis. To test this idea, we measured binding of a fluorescent ATP analog to recombinantly expressed and purified WT and the V95E mutant proteins and found that, indeed, both proteins had similar affinity (WT Kd 350 ± 68 nM; V95E Kd 470 ± 120 nM; Supplementary Figure S7). Likewise, when we measured steady state ATP hydrolysis in malachite green assays, the WT and V95E proteins had very similar initial velocity (V_0_) rates (~16 pmol ATP/μM chaperone/min), demonstrating that the V95E mutation does not meaningfully affect the basal enzymatic activity of HSC70.

Next, we tested whether the V95E mutation might impact collaboration with co-chaperones. When we titrated DnaJB4 into hydrolysis reactions containing recombinantly purified HSC70, we found that the ATPase activities of both WT and V95E were stimulated (Figure 7B; left). By fitting the results to the Michalis-Menten equation, we derived pseudo-Km values, showing that the apparent affinities for DnaJB4 were comparable (WT = 0.092 ± 0.018 μM; V95E = 0.088 ± 0.022 μM). We did notice a modest decrease in the pseudo-V_max_, with WT having a maximum hydrolysis rate of 42.90 ± 0.365 μmol P_i_/min, and V95E having a pseudo-V_max_ of 36.63 ± 1.41 μmol P_i_/min (^***^p = 0.0001). Taken together, these data suggest that V95E HSC70 has a modest effect on DnaJB4-stimulated hydrolysis of ATP. Next, we explored the response of WT and V95E to a representative of the NEF family of co-chaperones: BAG1. In these experiments, BAG1 was titrated into a mixture of HSC70 (1 μM) and DnaJB4 (0.1 μM). The results revealed that BAG1 stimulates WT HSC70’s ATPase activity, but that V95E was relatively insensitive to BAG1 (Figure 7B; right), only reaching approximately 25% stimulation at the highest concentration tested. These data suggest that V95E HSC70 has perturbed interactions with co-chaperones, especially BAG1. We next tested the effects of V95E on a more complex chaperone function: refolding of the denatured client. In these assays, chemically denatured *Renilla* luciferase is added to a mixture of chaperones, co-chaperones, and ATP and the ability to refold the client protein is measured by production of light (luminescence). With either DnaJB4 (Figure 7C; left) or the combination of DnaJB4 and BAG1 (Figure 7C; right), both WT HSC70 and V95E HSC70 were able to refold luciferase to a similar extent. Thus, the effects of V95E on ATPase assays do not seem to dramatically impact refolding activity.

## 4 Discussion

In the present study, we established a novel rat model of NAD, F344-*kk*/*kk* rat strain, and identified a missense mutation (V95E) in the *Hspa8* gene of the rat model. We also developed *Hspa8*-KI rats carrying the V95E mutation and found the KI rats exhibited NAD. Thus, we considered that *Hspa8* is a causative gene for NAD in the F344-*kk*/*kk* rats. The *Hspa8* gene encodes a 70-kDa heat-shock cognate protein (HSC70) that is known as a chaperone. Because *Hspa8* gene mutation has not been known to cause any neurodegenerative diseases either in humans or animals, our result is the first evidence that HSPA8/HSC70 is directly involved in the pathogenesis of NAD.

The F344-*kk*/*kk* rats exhibited the hind limb gait abnormality and the axonal swelling in histopathology, also called the spheroid (Figure 1). Since the lower limb ataxia is a major clinical symptom of the NAD and the spheroid is a hallmark of the NAD (Bouley et al., 2006; Tanaka et al., 2017), we diagnosed F344-*kk*/*kk* rats as NAD. The spheroids distributed throughout the CNS and predominantly located in the sensory tracts associated with proprioceptive sense (deep sensation). Specifically, the spheroids located mainly in the fasciculus gracilis of the dorsal cord, and the posterior column nuclei and nucleus cuneatus accessorius of the medulla oblongata. Given the distribution of the spheroids, we considered that sensory feedback signals might be interrupted (sensory ataxia) (Chhetri et al., 2014; Zhang et al., 2021) and might cause progressive neurological symptoms including abnormal hind limb gaits in F344-*kk*/*kk* rats.

Hind limb gait abnormalities in F344-*kk*/*kk* rats may partly be similar to those of human hereditary spastic paraplegia (HSP). The HSP refers to a group of motor neurodegenerative disorders which involve slowly progressive lower limbs spasticity and muscle weakness (Fink, 2013; Elsayed et al., 2021; Murala et al., 2021). The histopathological features of HSP are axonal degeneration and they are often limited in the spinal cord, especially in the lateral corticospinal tract (the pyramidal motor system) (Fink, 2013). Due to the histopathological differences in the morphology and location, we considered that the F344-*kk*/*kk* rats exhibited the NAD. Since both HSP and NAD are heterogenous disorders, some HSP overlap with NAD. Specifically, some HSP patients carried the NAD-causative *PLA2G6* mutations (Ozes et al., 2017). *C19ORF12* gene can be responsible for both HSP and NAD (Hartig et al., 2011). Thus, there is the possibility that the *HSP8A* gene mutation will be found in the HSP patients and the potential that the *Hsp8a*-mutant rats will also be used as a model of HSP.

In neurodegenerative diseases, different structural and/or functional proteins accumulated in spheroids. In F344-*kk*/*kk* rats, electron-dense bodies, degenerated mitochondria, and membranous or tubular structures accumulated in the spheroids. Additionally, the spheroids were strongly positive for synaptophysin, APP, NFs, or ubiquitin (Figure 3). The accumulation of synaptophysin in the dystrophic axons may indicate the dysfunction of the synapse at the presynaptic portion. APP is transported along axons by fast anterograde transport (Gentleman et al., 1993; Hayashi et al., 2015). NFs and ubiquitin are transported over long distances via slow axonal transport (Bizzi et al., 1991). Abnormal accumulations of these proteins were not observed within normal axons. Thus, we considered that both slow and fast axonal transports would be impaired in the CNS of F344-*kk*/*kk* rats.

HSC70 has been known to be a molecular chaperone that has important roles in axonal transport (Terada et al., 2010). The axonal transport system consists of fast and slow transports. The fast transport system is driven by Kinesin-1 and the kinesin superfamily motor proteins and mainly transports membranous organelles. The slow transport system also uses the Kinesin-1 in a different manner than the fast system and transports cytoplasmic proteins. HSC70 can switch over between the fast and slow axonal transport through the DnaJ-like domain of the kinesin light chain (KLC) (Terada et al., 2010). In addition, inactivation of ATPase activity of HSC70 blocks the slow axonal transport and leads to the presynaptic accumulation of synapsin (Ganguly et al., 2017). These important roles of HSC70 in the axonal transport supported our thought that the axonal transport system would be impaired in F344-*kk*/*kk* rats. Such an impaired transport system may lead to the accumulation of various kinds of membrane organelles or protein complexes in the axons of the *Hspa8*-mutant F344-*kk*/*kk* rats. Moreover, HSC70 also has a critical role in chaperone-mediated autophagy (CMA) (Bonam et al., 2019). HSC70 recognizes substrates to be processed and incorporates them into lysosomes. Abnormal HSC70 or LAMP2A (lysosomal-associated membrane protein 2A) expression and CMA activity have been implicated in the pathogenesis of neurodegenerative diseases such as amyotrophic lateral sclerosis and Parkinson’s disease (Coyne et al., 2017; Sirtori et al., 2020) It is possible that CMA might be also impaired in the nervous systems of F344-*kk*/*kk* rats.

To explore possible molecular mechanisms, we compared *in vitro* chaperone functions of the V95E mutant HSC70 to those of the WT. We found that the V95E mutant HSC70 was largely normal in its intrinsic ATP binding and hydrolysis. The V95E HSC70 did not seem to dramatically impact the client refolding activity which was assayed by the luciferase refolding experiments (Figure 7C). However, we did observe a reduced ATPase activity of the V95E HSC70 protein when stimulated with the co-chaperones DnaJB4 and BAG1 (Figure 7B). In addition, the expression level of the V95E HSC70 was significantly decreased in the spinal cord of *Hspa8*-KI homozygous rats compared with the WT rats (Figure 6). These findings suggest that the reduced ATPase activity in V95E HSC70 may be enhanced in the *Hspa8*-mutant rats. The V95E amino acid alteration in this housekeeping protein is likely to be enough to cause the axonal swelling without detrimental effects on animal viability.

For axonal transport, the DnaJ-like domain of the KLC has a crucial role, binding with HSC70 and in the switchover between slow and fast transports (Terada et al., 2010). These findings provided us with an attractive hypothesis that the V95E HSC70 mutant might fail to fully couple with the DnaJ-like domain of KLC and thereby axonal transport may be impaired in the axonal spheroids of F344-*kk*/*kk* rats.

In summary, we developed a novel rat model of NAD, F344-*kk*/*kk* rats. Histopathological analyses suggest they have deficits in axonal transport. The causative mutation of NAD was the V95E missense mutation of the *Hspa8* gene that encoded a chaperone protein HSC70. The V95E mutant HSC70 protein exhibited reduced ATPase activity when stimulated by the co-chaperons DnaJB4 and BAG1. HSPA8/HSC70 is a constitutively expressed chaperone protein that is essential for keeping biological function, thus even subtle damage to the biochemical function may cause the severe phenotype in rats. Further studies on the *Hspa8*-mutant rats will allow us to discover novel mechanisms underlying the development and progression of NAD and axonal degeneration.

## Data availability statement

The raw data supporting the conclusions of this article will be made available by the authors, without undue reservation.

## Ethics statement

The animal study was approved by Animal Research Committees of Kyoto University, Osaka Metropolitan University, and Tokyo University of Agriculture. The study was conducted in accordance with the local legislation and institutional requirements.

## Author contributions

MT: Investigation, Writing – original draft, Conceptualization, Data curation, Formal analysis, Methodology, Resources. RF: Investigation, Writing – review. TSek: Investigation, Writing – review & editing. JH: Investigation, Writing – review & editing. OJ: Investigation, Writing – review & editing. DT: Investigation, Writing – review & editing. KK: Resources, Writing – review & editing. TSer: Writing – review & editing. HH: Investigation, Writing – review & editing. KH: Investigation, Resources, Writing – review & editing. TM: Writing – review & editing. MK: Writing – review & editing. JG: Writing – original draft, Conceptualization, Data curation, Formal analysis, Methodology. TK: Conceptualization, Data curation, Formal analysis, Resources, Supervision, Writing – original draft.
