# Supplementary material for: A missense mutation in the Hspa8 gene encoding heat shock cognate protein 70 causes neuroaxonal dystrophy in rats
Source: Front Neurosci. 2024 Feb 6;18:1263724. doi: 10.3389/fnins.2024.1263724 (PMC10880117; doi:10.3389/fnins.2024.1263724)
Supplement: Supplementary file 1 [file Table_1.pdf]

Supplementary Materials

A missense mutation in the Hspa8 gene encoding heat shock cognate protein 70 causes neuroaxonal dystrophy in rats

Miyuu Tanaka, Ryoko Fujikawa, Takahiro Sekiguchi, Oleta T. Johnson, Jason Hernandez, Daisuke Tanaka, Kenta Kumafuji, Tadao Serikawa, Hieu Hoang Trung, Kosuke Hattori, Tomoji Mashimo, Mitsuru Kuwamura, Jason E. Gestwicki, Takashi Kuramoto*

*** Correspondence:** Corresponding Author: Takashi Kuramoto, Ph.D.

email: tk206782@nodai.ac.jp

Supplementary Figures

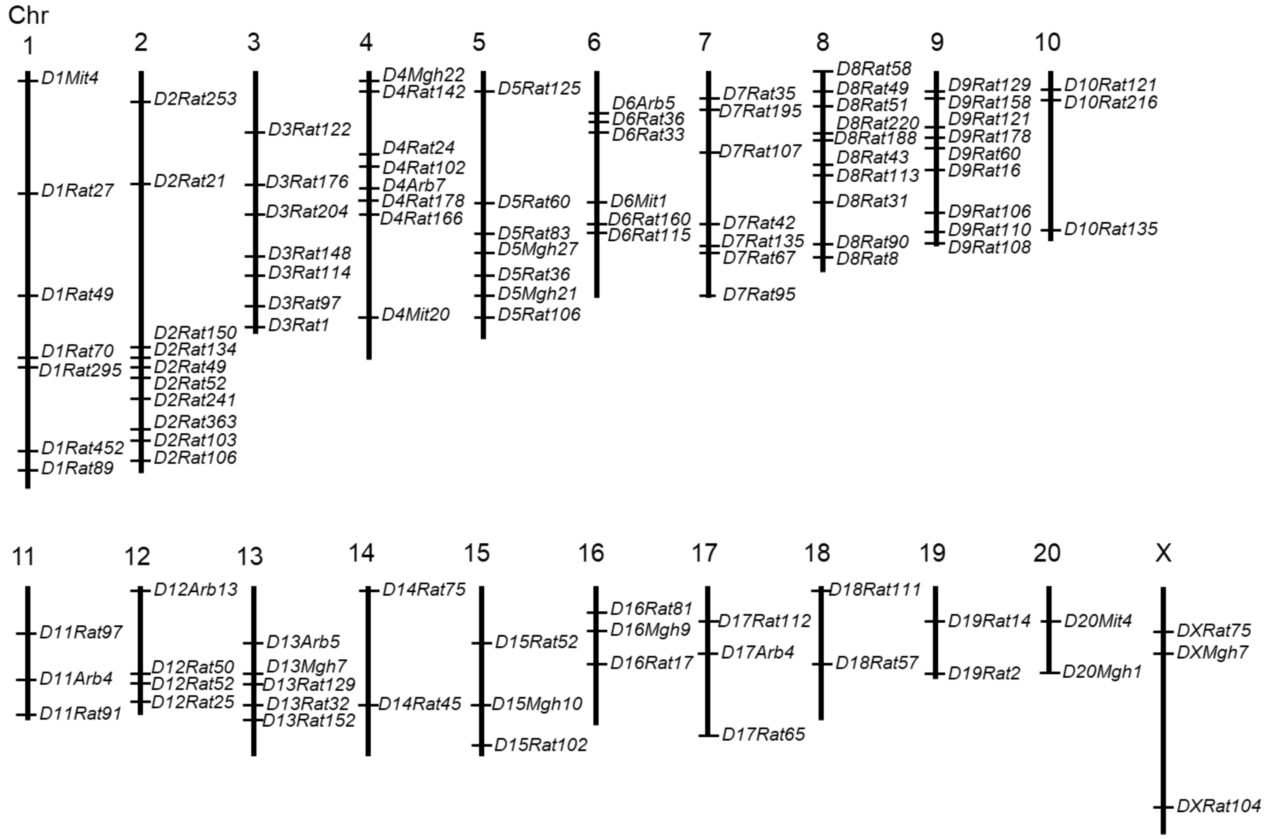

Supplementary Figure 1. Chromosomal locations of the 106 simple sequence length polymorphism (SSLP) markers used in this study.

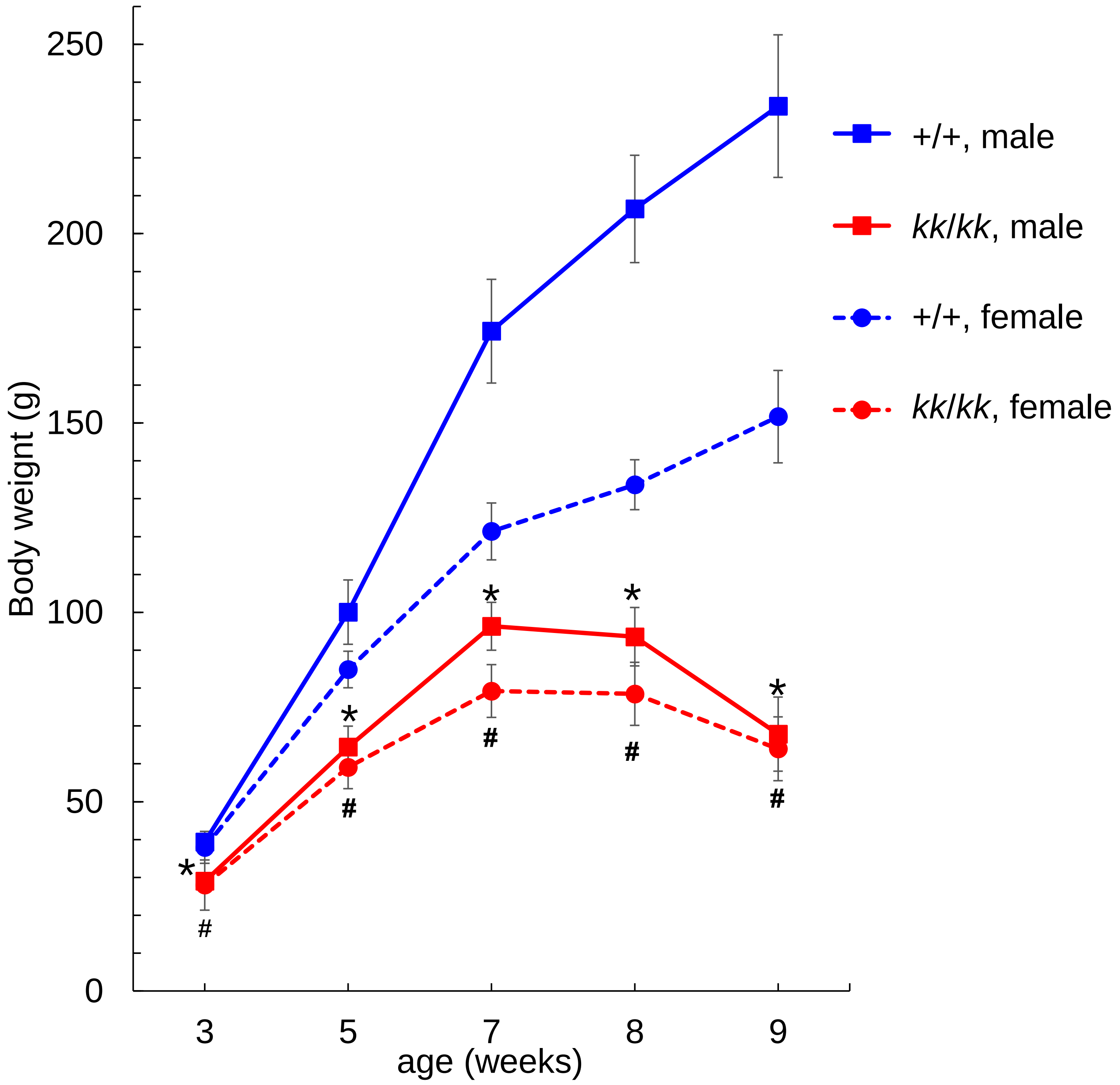

Supplementary Figure 2. Temporal changes in body weight of the F344-*kk*/*kk* rats. Blue lines: wild-type (+/+) and Red lines; homozygous (*kk*/*kk*) rats. 3 weeks of age; +/+, male (n = 9), *kk*/*kk*, male (n = 6), +/+, female (n = 6) and *kk*/*kk*, female (n = 3). 5 weeks of age; +/+, male (n = 9), *kk*/*kk*, male (n = 8), +/+, female (n = 9) and *kk*/*kk*, female (n = 7). 7 weeks of age; +/+, male (n = 9), *kk*/*kk*, male (n = 8), +/+, female (n = 9) and *kk*/*kk*, female (n = 7). 8 weeks of age; +/+, male (n = 9), *kk*/*kk*, male (n = 8), +/+, female (n = 9) and *kk*/*kk*, female (n = 9). 9 weeks of age; +/+, male (n = 9), *kk*/*kk*, male (n = 12), +/+, female (n = 9) and *kk*/*kk*, female (n = 7). $^{*}P < 0.05$ (male) and $^{\#}P < 0.05$ (female) by Welch's t-test compared with the wild-type rats.

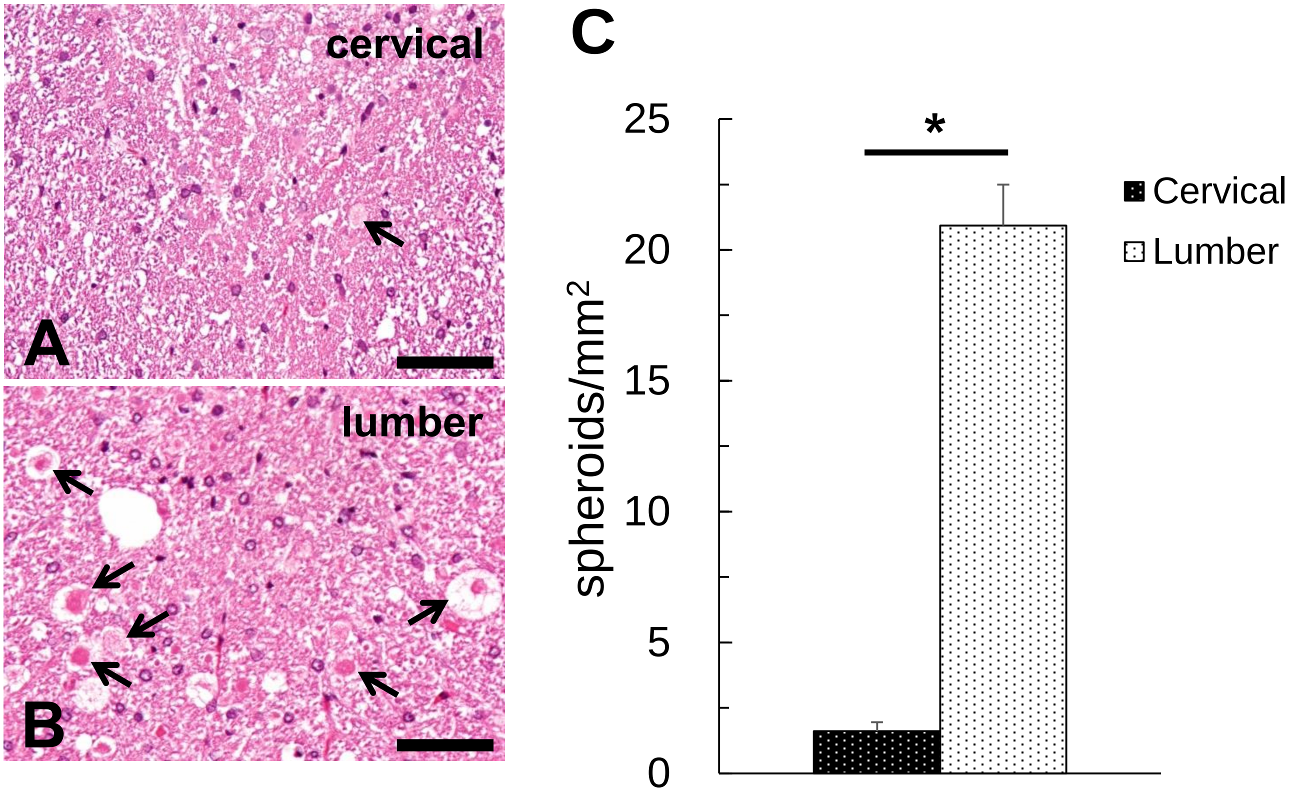

Supplementary Figure 3. Histopathology **(A and B)** and the number of spheroids **(C)** in the dorsal cord of the cervical **(A)** and lumber **(B)** spinal cord in the *kk/kk* homozygous rats at 10 weeks of age. Arrows indicate axonal spheroids. HE. Bars: 50 µm. The number of spheroids is significantly increased in the lumber spinal cord compared with the cervical spinal cord. Data are presented as the number of spheroids/mm^2 (n = 5 in each group). *$P < 0.05$ by Welch's t-test.

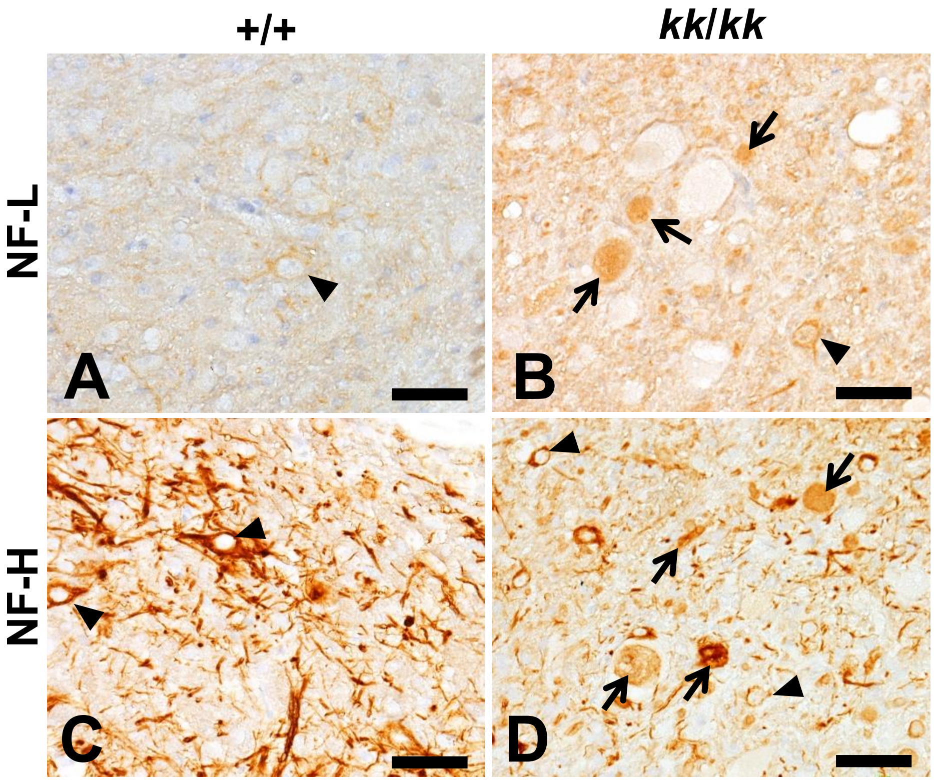

Supplementary Figure 4. IHC for NF-L **(A and B)** and NF-H **(C and B)** of the medulla oblongata of the wild-type (+/+) and *kk*/*kk* homozygous rat at 10 weeks of age. Arrows indicate swollen axons. Arrowheads: neurons. Bars: 50 µm.

A

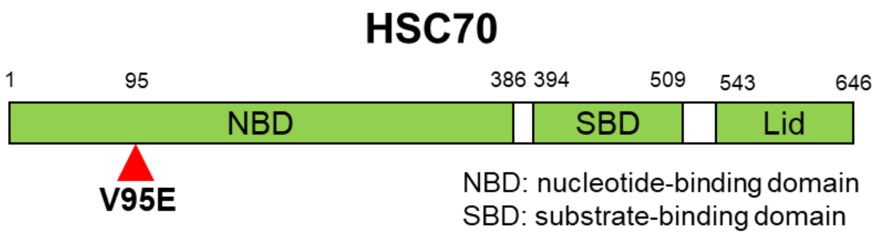

B

```
hHSPA8_protein_NP_006588    1:MSKGPAVGIDLGTTYSCVGVFQHGKVEIIANDQGNRTTPSYVAFTDTERLIGDAAKNQVA 60
mHSPA8_protein_NP_111244    1:MSKGPAVGIDLGTTYSCVGVFQHGKVEIIANDQGNRTTPSYVAFTDTERLIGDAAKNQVA 60
rHSPA8_protein_NP_077327    1:MSKGPAVGIDLGTTYSCVGVFQHGKVEIIANDQGNRTTPSYVAFTDTERLIGDAAKNQVA 60
                              ************************************************************

                                                                   95
hHSPA8_protein_NP_006588   61:MNPTNTVFDAKRLIGRRFDDAVVQSDMKHWPFMVVNDAGRPKVQVEYKGETKSFYPEEVS 120
mHSPA8_protein_NP_111244   61:MNPTNTVFDAKRLIGRRFDDAVVQSDMKHWPFMVVNDAGRPKVQVEYKGETKSFYPEEVS 120
rHSPA8_protein_NP_077327   61:MNPTNTVFDAKRLIGRRFDDAVVQSDMKHWPFMVVNDAGRPKVQVEYKGETKSFYPEEVS 120
                              ************************************************************

hHSPA8_protein_NP_006588  121:SMVLTKMKEIAEAYLGKTVTNAVVTVPAYFNDSQRQATKDAGTIAGLNVLRIINEPTAAA 180
mHSPA8_protein_NP_111244  121:SMVLTKMKEIAEAYLGKTVTNAVVTVPAYFNDSQRQATKDAGTIAGLNVLRIINEPTAAA 180
rHSPA8_protein_NP_077327  121:SMVLTKMKEIAEAYLGKTVTNAVVTVPAYFNDSQRQATKDAGTIAGLNVLRIINEPTAAA 180
                              ************************************************************

hHSPA8_protein_NP_006588  181:IAYGLDKKVGAERNVLIFDLGGGTFDVSILTIEDGIFEVKSTAGDTHLGGEDFDNRMVNH 240
mHSPA8_protein_NP_111244  181:IAYGLDKKVGAERNVLIFDLGGGTFDVSILTIEDGIFEVKSTAGDTHLGGEDFDNRMVNH 240
rHSPA8_protein_NP_077327  181:IAYGLDKKVGAERNVLIFDLGGGTFDVSILTIEDGIFEVKSTAGDTHLGGEDFDNRMVNH 240
                              ************************************************************

hHSPA8_protein_NP_006588  241:FIAEFKRKHKKDISENKRAVRRLRTACERAKRTLSSSTQASIEIDSLYEGIDFYTSITRA 300
mHSPA8_protein_NP_111244  241:FIAEFKRKHKKDISENKRAVRRLRTACERAKRTLSSSTQASIEIDSLYEGIDFYTSITRA 300
rHSPA8_protein_NP_077327  241:FIAEFKRKHKKDISENKRAVRRLRTACERAKRTLSSSTQASIEIDSLYEGIDFYTSITRA 300
                              ************************************************************

hHSPA8_protein_NP_006588  301:RFEELNADLFRGTLDPVEKALRDAKLDKSQIHDIVLVGGSTRIPKIQKLLQDFFNGKELN 360
mHSPA8_protein_NP_111244  301:RFEELNADLFRGTLDPVEKALRDAKLDKSQIHDIVLVGGSTRIPKIQKLLQDFFNGKELN 360
rHSPA8_protein_NP_077327  301:RFEELNADLFRGTLDPVEKALRDAKLDKSQIHDIVLVGGSTRIPKIQKLLQDFFNGKELN 360
                              ************************************************************

hHSPA8_protein_NP_006588  361:KSINPDEAVAYGAAVQAAILSGDKSENVQDLLLLDVTPLSLGIETAGGVMTVLIKRNTTI 420
mHSPA8_protein_NP_111244  361:KSINPDEAVAYGAAVQAAILSGDKSENVQDLLLLDVTPLSLGIETAGGVMTVLIKRNTTI 420
rHSPA8_protein_NP_077327  361:KSINPDEAVAYGAAVQAAILSGDKSENVQDLLLLDVTPLSLGIETAGGVMTVLIKRNTTI 420
                              ************************************************************

hHSPA8_protein_NP_006588  421:PTKQTQTFTTYSDNQPGVLIQVYEGERAMTKDNNLLGKFELTGIPPAPRGVPQIEVTFDI 480
mHSPA8_protein_NP_111244  421:PTKQTQTFTTYSDNQPGVLIQVYEGERAMTKDNNLLGKFELTGIPPAPRGVPQIEVTFDI 480
rHSPA8_protein_NP_077327  421:PTKQTQTFTTYSDNQPGVLIQVYEGERAMTKDNNLLGKFELTGIPPAPRGVPQIEVTFDI 480
                              ************************************************************

hHSPA8_protein_NP_006588  481:DANGILNVSAVDKSTGKENKITITNDKGRLSKEDIERMVQEAEKYKAEDEKQRDKVSSKN 540
mHSPA8_protein_NP_111244  481:DANGILNVSAVDKSTGKENKITITNDKGRLSKEDIERMVQEAEKYKAEDEKQRDKVSSKN 540
rHSPA8_protein_NP_077327  481:DANGILNVSAVDKSTGKENKITITNDKGRLSKEDIERMVQEAEKYKAEDEKQRDKVSSKN 540
                              ************************************************************

hHSPA8_protein_NP_006588  541:SLESYAFNMKATVEDEKLQGKINDEDKQKILDKCNEIINWLDKNQTAEKEEFEHQQKELE 600
mHSPA8_protein_NP_111244  541:SLESYAFNMKATVEDEKLQGKINDEDKQKILDKCNEIISWLDKNQTAEKEEFEHQQKELE 600
rHSPA8_protein_NP_077327  541:SLESYAFNMKATVEDEKLQGKINDEDKQKILDKCNEIISWLDKNQTAEKEEFEHQQKELE 600
                              **************************************.*********************

hHSPA8_protein_NP_006588  601:KVCNPIITKLYQSAGGMPGGMPGGFPGGGAPPSGGASSGPTIEEVD               646
mHSPA8_protein_NP_111244  601:KVCNPIITKLYQSAGGMPGGMPGGFPGGGAPPSGGASSGPTIEEVD               646
rHSPA8_protein_NP_077327  601:KVCNPIITKLYQSAGGMPGGMPGGFPGGGAPPSGGASSGPTIEEVD               646
                              **********************************************
```

Supplementary Figure 5. Schematic representation of functional domains and positions of the mutation of HSPA8/HSC70. **(A)** Schematic representations of rat HSC70 protein. The V95E mutation of F344-*kk*/*kk* rats is located in the nucleotide-binding domain (NBD). **(B)** The comparison of HSC70 amino acid sequences among humans (h), mice (m), and rats (r). The amino acid sequence of HSC70 is highly conserved among species.

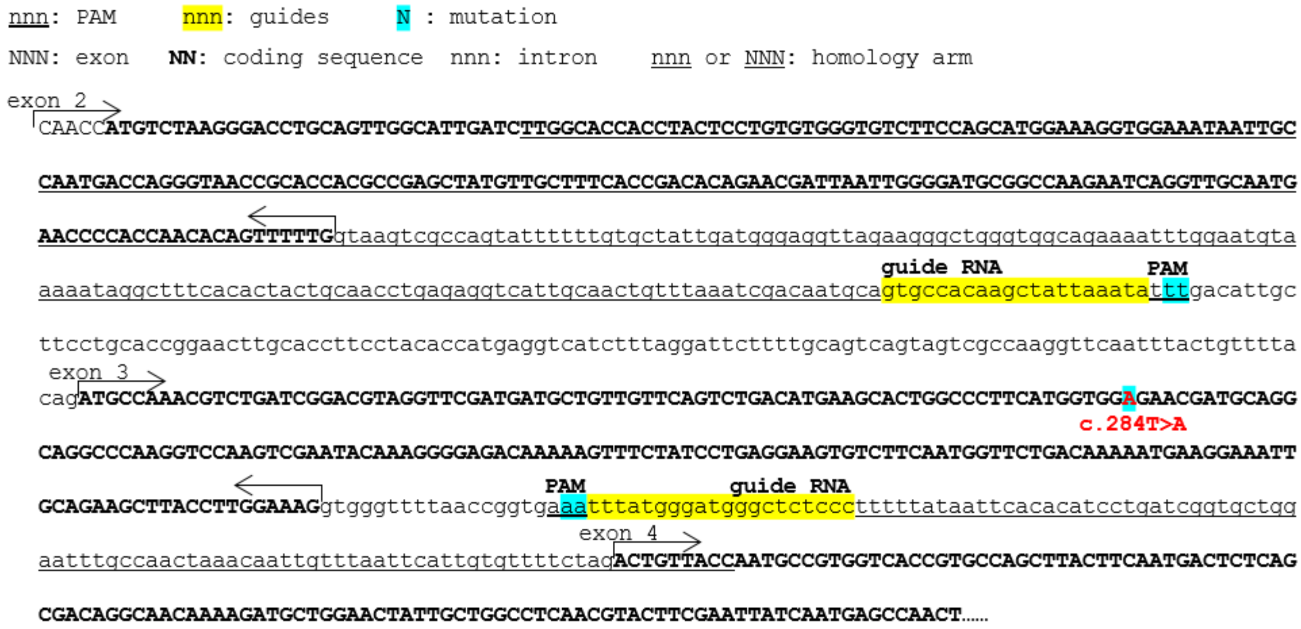

Supplementary Figure 6. Development of *Hspa8*-KI rats

Positions of guide RNAs and genomic region covered with lsODN to develop *Hspa8*-KI rats. Exons 2–4 and introns of the rat *Hspa8* gene are represented in upper and lower case, respectively. The coding sequence is represented in bold. The guide RNAs (highlighted in yellow) were designed on the intron 2 and 3 sequences, and the PAM sequences are indicated by double lines. The LsODN carrying the *kk* mutation (A; highlighted in red) was 768-bp in length including 309-bp and 85-bp homology arms which are underlined.

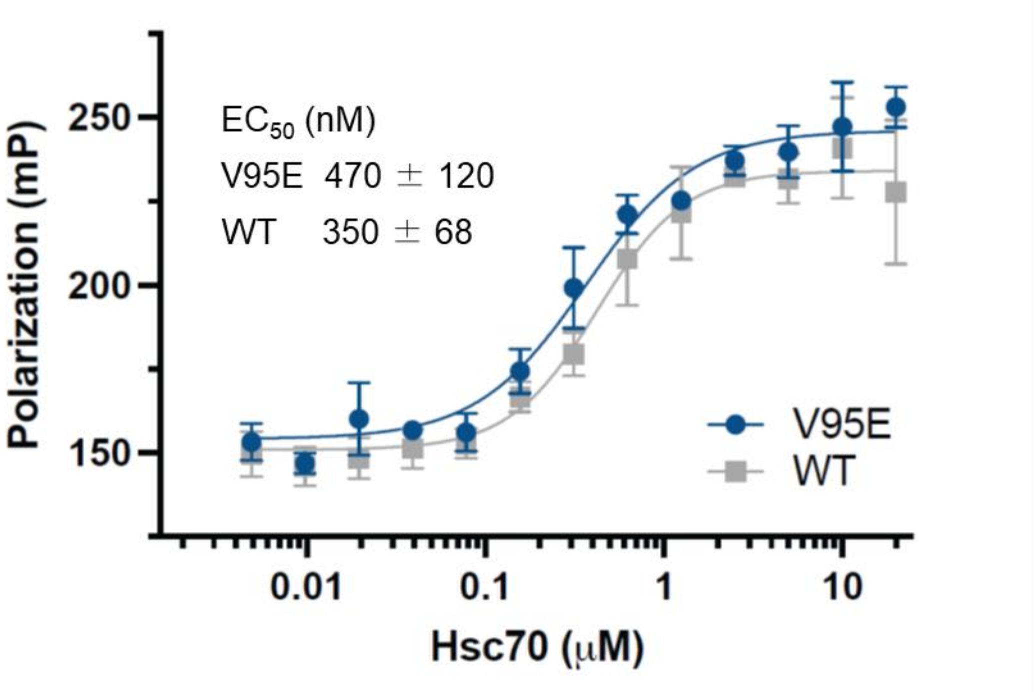

Supplementary Figure 7. Additional biochemical studies to explore the differences between WT and V95E HSC70. Fluorescence polarization (FP) was used to measure HSC70 binding to a fluorescent ATP. Both WT and mutant V95E had similar binding affinity (~400 nM). Data are expressed as mean ± standard deviation (SD).

Supplementary Tables

Supplementary Table 1. Primary antibodies used in this study

Antibody	Host species	Clone	Dilution	Antigen retrieval	Manufacturer
Synaptophysin	Mouse	SY38	1:2,000	None	Merck Millipore, CA, USA
Ubiquitin	Mouse	Ubi-1	1:25,000	Microwave in Citrate buffer (pH 6.0), 20 min	Merck Millipore, CA, USA
Amyloid β precursor protein (APP)	Mouse	2C11	1:1,000	Microwave in Citrate buffer (pH 6.0), 20 min	Merck Millipore, CA, USA
Neurofilament light chain (NF-L)	Mouse	DA2	1:1,000	Microwave in Tris-EDTA buffer (pH 9.0), 20 min	Cell Signaling Technology, MA, USA
Neurofilament heavy chain (NF-H)	Mouse	RMdO20	1:1,000	Microwave in Citrate buffer (pH 6.0), 20 min	Cell Signaling Technology, MA, USA

Supplementary Table 2. Primers for RT-PCR and sequencing

Primer name	Sequence (5′ > 3′)
r*Hspa8*-1	GGGTCTCTGTGTGGTCTCGT
r*Hspa8*-2	CAGCAGCAGTTGGCTCATT
r*Hspa8*-3	GTCACCGTGCCAGCTTACTT
r*Hspa8*-4	CCACCCACCAGGACAATATC
r*Hspa8*-5	TGAGGAGTTGAATGCTGACCT
r*Hspa8*-6	TCTTGTTCTCCTTTCCTGTGC
r*Hspa8*-7	CAACAACCTGCTTGGGAAGT
r*Hspa8*-8	TTTATTATTCCATGTTACTTGTTTTGG
