# Supplementary material for: A missense mutation in the Hspa8 gene encoding heat shock cognate protein 70 causes neuroaxonal dystrophy in rats
Source: Front Neurosci. 2024 Feb 6;18:1263724. doi: 10.3389/fnins.2024.1263724 (PMC10880117; doi:10.3389/fnins.2024.1263724)
Supplement: Supplementary file 2 [file Presentation_1.PPTX]

## Slide 1
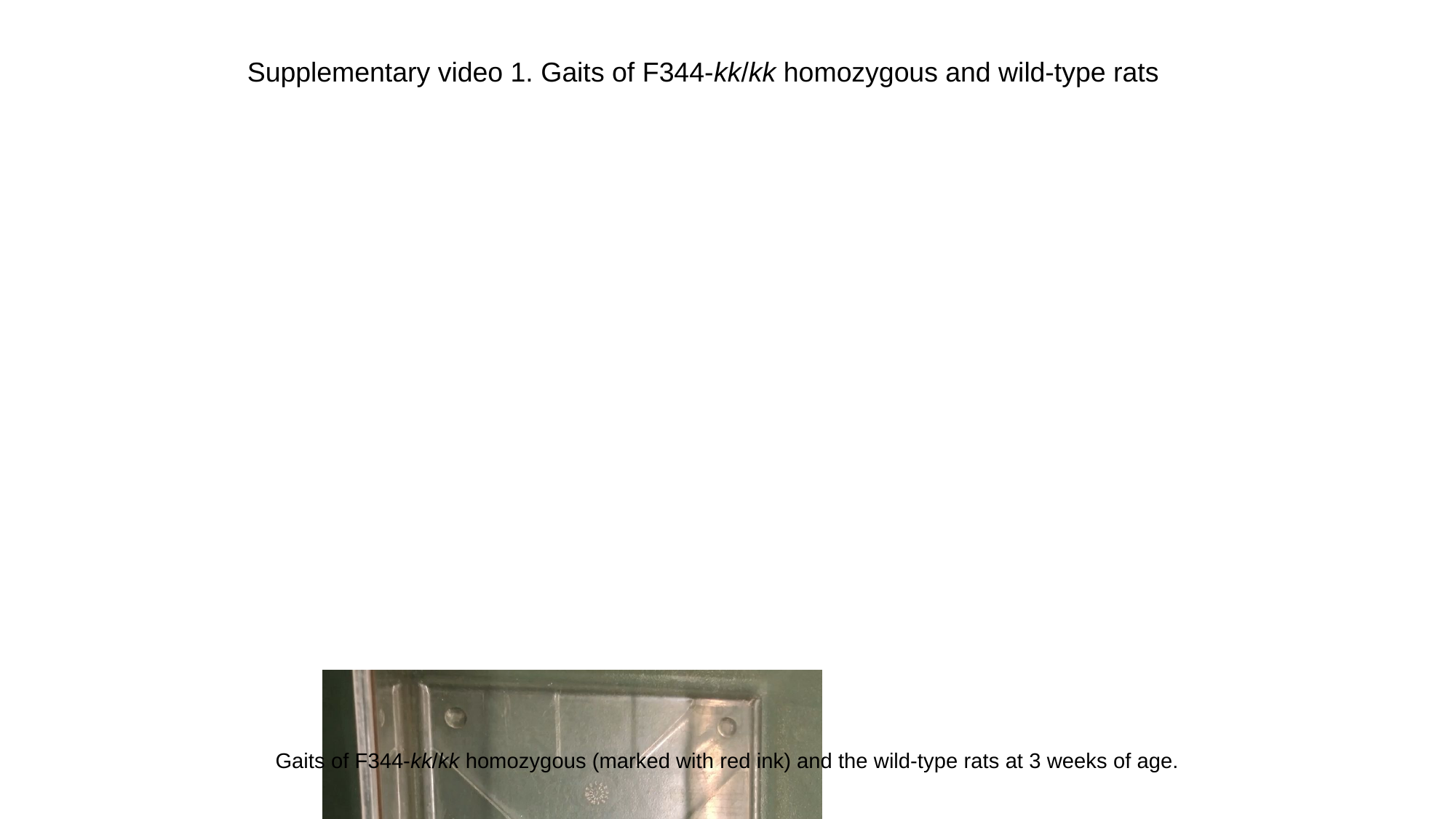

Supplementary video 1. Gaits of F344-kk/kk homozygous and wild-type rats
Gaits of F344-kk/kk homozygous (marked with red ink) and the wild-type rats at 3 weeks of age.
